# Commentary: The Experience of Depression during Careers of Elite Male Athletes

**DOI:** 10.3389/fpsyg.2016.01609

**Published:** 2016-10-20

**Authors:** Monna Arvinen-Barrow

**Affiliations:** Department of Kinesiology, Integrative Health Care and Performance, University of Wisconsin-MilwaukeeMilwaukee, WI, USA

**Keywords:** male, depression, elite sport culture, identity, masculinity, commentary

As I am writing this commentary on “The Experience of Depression during Careers of Elite Male Athletes” by Doherty et al. (2016) concurrently with the 2016 Olympics in Rio, the topicality of the paper is felicitous. A number of prominent Olympians, both male and female, have openly come out in the popular media and discussed their personal battles with depression, including English Gardener (USA; track & field), Jack Green (UK; track & field), Michael Phelps (USA; swimming), and Allison Schmitt (USA; swimming) to name a few (Frank, 2016; Murphy, 2016; Powers, 2016; Schnell, 2016). These personal accounts highlight the importance of recognizing that elite athletes, who, like the population at large, are not immune to mental health problems (Rice et al., 2016).

The article by Doherty et al. (2016) is the first of its kind to truly delve into retrospective experiences of athletes who had experienced depression during their sporting careers. By adopting a qualitative research design, and using both descriptive and interpretative analysis (Elliot and Timulak, 2005), the authors have cleverly chosen a methodology that enables the exploration of a phenomenon from the participant perspective. This allowed the emergence of data in relation to the nature and defining features of depression in male elite athletes, as well as detailed exploration of “how the culture of sport and the interplay between the athletes' sense of self and the elite performance environment influenced how they experience, expressed, and responded to depression during their careers” (Doherty et al., 2016). As such, the authors were able to explore an under researched area in greater depth, and to provide commendable and meaningful insights into the phenomenon.

One of the key findings worthy of further attention is the role of identity in depression. Athletic identity, which according to Horton and Mack (2000) is considered to be both a cognitive structure (e.g., “I am athletic”) and a social role (e.g., “I am a swimmer”) and is typically associated with positive impressions of oneself. However, the current study suggested that athletic identity can influence the emergence, manifestation, development, and the adaptive and maladaptive processes of recovery from depression. More specifically, Doherty et al. (2016) found that having a strong athletic identity and performance pressures made athletes more vulnerable for depression, and enabled the masking of depressive symptoms. Identity also influenced the process of recovery, as it was partially characterized by shifts in identity toward more multidimensional sense of self.

When placed in the wider social context of sport, it is easy to see how athletic identity development among elite male athletes can predispose an individual to depression. Internal and external expectations of personal characteristics such as masculinity, mental toughness, and grit are likely to contribute the development of a more unidimensional identity (Young and White, 2000; Jones et al., 2002; Duckworth et al., 2007). One does not have to look far to see how the above characteristics are differently constructed for male and female athletes (e.g., Rogers, 2016). Male athletes are often referred in the popular and social media as strong, powerful, and fast. In contrast, female athlete media coverage includes explicit references to other social identities they possess: wife, fiancée, mother, grandmother, and both of the above portrayals have also sparked a lot of social media interest and controversy. Although the little feminist in me finds the apparent differences in male and female portrayals offensive and sexist, the mental health professional in me cannot help but wonder: How do popular and social media contribute to the development of athlete's multi/unidimensional identities of self? To what extent does popular and social media and its portrayal of male and female athletes contribute to the occurrence and manifestation of, and recovery from depression? Moreover, how does athlete's own engagement and interaction with different forms of media influence their affect and ultimately their mental health?

Given that research in the area of elite athlete depression is sparse, further high-quality research is certainly warranted. Thus far, existing literature has highlighted significant major life events, sport injury, and chronic stress as potential risk factors for depression and other mental health problems (Rice et al., 2016) particularly when an athletes' sense of self is strongly tied into being an athlete. It is therefore important that the professionals working with elite athletes “know their athletes” both in sport and out of their immediate sporting environment, and have an understanding of events that might act as a catalyst for the emergence of depression. Building on Doherty et al. (2016) work, I would particularly welcome more phenomenological research investigating elite athlete depression from a more interprofessional and biopsychosocial perspective. After all, depression is typically characterized by physiological (e.g., loss of energy, appetite, inability to sleep), and psychological (e.g., range of debilitative cognitive appraisals, negative emotions, and/or changes in typical behaviors) maladaptive changes in the body. Moreover, depression typically affects, and is affected by, number of personal (e.g., genetics, age) and social factors (e.g., environment, climate, professionals working with the athlete).

In summary, I am honored to comment on this article and applaud the authors for addressing such an important, yet hugely under attended topic. I find the article to be an important addition to the elite athlete depression literature. From building a rationale, to aims, design, data analysis, and interpretation of the findings, the authors are able to capture athletes own accounts of experiencing depression, and pave the way for further important research in this field. Looking forward to more to come.

## Author contributions

MAB is solely responsible for the conception or design of the work; and drafting the work or revising it critically for important intellectual content; and final approval of the version to be published; and agreeing to be accountable for all aspects of the work in ensuring that questions related to the accuracy or integrity of any part of the work are appropriately investigated and resolved.

### Conflict of interest statement

The author declares that the research was conducted in the absence of any commercial or financial relationships that could be construed as a potential conflict of interest.
